# Age-stratified gut microbial changes in diarrheal calves: insights from 16S rRNA sequencing across early development

**DOI:** 10.3389/fmicb.2025.1633162

**Published:** 2025-09-15

**Authors:** Yanli Zhang, Chunfu Li, Luyang Tang, Fenqi Li, Xuanrong Fu, Yu Hao, Jian Li, Xinyu Feng, Wei Hu

**Affiliations:** ^1^College of Life Sciences, Inner Mongolia University, Hohhot, China; ^2^Basic Medicine College, Guangxi Traditional Chinese Medical University, Nanning, China; ^3^School of Global Health, Chinese Center for Tropical Diseases Research, Shanghai Jiao Tong University School of Medicine, Shanghai, China; ^4^One Health Center, Shanghai Jiao Tong University-The University of Edinburgh, Shanghai, China; ^5^Department of Infectious Diseases, Huashan Hospital, State Key Laboratory of Genetic Engineering, Ministry of Education Key Laboratory for Biodiversity Science and Ecological Engineering, Ministry of Education Key Laboratory of Contemporary Anthropology, School of Life Sciences, Fudan University, Shanghai, China

**Keywords:** neonatal diarrhea, calf, gut microbiota, developmental dysbiosis, microbial networks

## Abstract

**Introduction:**

Neonatal calf diarrhea (NCD) remains a leading cause of mortality in calves under 1 month, yet how gut microbial responses vary across developmental stages remains unexplored. This study investigates age-stratified microbiome dynamics during NCD to enable precision interventions.

**Materials and methods:**

This study investigated 60 female Holstein calves (1, 21, and 30 days old) from a commercial dairy farm, equally divided between healthy and diarrheal groups based on standardized fecal scoring. Fecal samples were collected aseptically, flash-frozen, and processed for 16S rRNA gene sequencing (V3-V4 region) using Illumina NovaSeq. Bioinformatics analyses included DADA2 pipeline for ASV calling, SILVA 138 database for taxonomic annotation, ANCOM-BC2 for differential abundance analysis (FDR < 0.05), PICRUSt2 for functional prediction, and SparCC networks (|r| > 0.6, *p* < 0.001) with Gephi visualization. Multivariate statistics, including PERMANOVA and canonical correspondence analysis were performed in QIIME2 and R (phyloseq/vegan packages), with all analyses rarefied to 39,161 sequences/sample.

**Results:**

The gut microbiome exhibited age-dependent succession, transitioning from Pseudomonadota dominance (47.2 ± 0.7%) at day 1 to Bacillota/Bacteroidota co-dominance (85.5 ± 8.2%) by day 30. Age explained significantly more compositional variance than diarrhea status (3.68% vs. 1.96%, *p* < 0.001). Three distinct age-specific diarrheal patterns emerged: (1) Early-stage (1-day-old) showed Bacillota/Pseudomonadota imbalances (84% of differential ASVs) with reduced network complexity (total node count, total edge count, average degree and modularity); (2) Mid-lactation (21-day-old) featured *Kurthia* as both significantly enriched (log2FC = 5.32) and a network hub (degree = 14); (3) Mature microbiota (30-day-old) displayed complex multi-phylum dysbiosis involving 10 metabolic pathways. *Clostridia_UCG-014* persisted across diarrheal networks, while healthy calves showed age-progressive increases in microbial connectivity (edges: 125 to 1,104). Only 2 ASVs demonstrated consistent differential abundance across age groups, confirming the temporal specificity of diarrheal dysbiosis.

**Conclusion:**

NCD-associated dysbiosis progresses through distinct developmental phases, from resilient phylum-level shifts in neonates to complex network disruptions in mature microbiota. The identification of stage-specific biomarkers (e.g., day 21 *Kurthia*) opens new avenues for age-tailored probiotic therapies and early intervention strategies.

## 1 Introduction

Neonatal calf diarrhea (NCD) is a major challenge in calf rearing, representing the leading cause of mortality in calves under 1 month of age, with direct mortality rates exceeding 50% (Agnol et al., 2021; Urie et al., 2018). Beyond acute losses, NCD exerts long-term detrimental effects on calf development, including reduced weight gain (Windeyer et al., 2014; Anderson et al., 2003), delayed age at first conception (Aghakeshmiri et al., 2017; Abuelo et al., 2021; Goh et al., 2024), and decreased milk yield in the first lactation (Heinrichs et al., 2005; Abuelo et al., 2021), ultimately compromising herd productivity. While antibiotic therapy remains a primary treatment for NCD (Urie et al., 2018), its overuse contributes to antimicrobial resistance, drug residues in animal products, and disruption of ruminal and intestinal microbiota, impairing feed efficiency and growth performance (Ji et al., 2018; Oultram et al., 2015; Pereira et al., 2016). These limitations underscore the urgent need for alternative strategies targeting the gut microbiome, a key player in NCD pathogenesis.

The early-life gut microbiota of calves undergoes rapid ecological succession, with richness and diversity increasing progressively during the first month (Klein-Jöebstl et al., 2019; Huang et al., 2024; Claus-Walker et al., 2024; Badman et al., 2019). Compositionally, the microbiome shifts from a Pseudomonadota (synonym Proteobacteria; Oren and Garrity, 2021)-dominated state (>60%) in neonates to a Bacillota (synonym Firmicutes; Oren and Garrity, 2021) and Bacteroidota co-dominated structure (collectively >50%) in older calves (Huang et al., 2024; Malmuthuge et al., 2015; Uyeno et al., 2010). Longitudinal studies delineate two dynamic phases: a colonization phase (0–3 days) and a stabilization phase (post-7 days) (Huang et al., 2024), with Pan et al. (2024) further demonstrating an age-dependent transition in community assembly—from stochastic-driven in early life to deterministic-driven after 3 weeks. NCD is associated with microbial ecosystem disruptions in calves (Ku et al., 2025; Kwon et al., 2021). Specific microbiota alterations emerge before clinical symptom manifestation (Chen et al., 2022; Jessop et al., 2024; Ma et al., 2020). However, research on whether NCD differentially impact these age-dynamic microbial communities remains scarce.

Harnessing microbial interventions has emerged as a promising paradigm. Probiotics (e.g., *Lactobacillus*, *Saccharomyces*) significantly reduce NCD incidence by enhancing intestinal homeostasis, enriching antimicrobial metabolite-producing symbionts, and modulating immunity (Wu et al., 2021), while also shortening diarrheal duration in affected calves (Renaud et al., 2019). Similarly, microbiota transplantation—transferring fecal microbes from healthy donors—restores gut microbial architecture and alleviates symptoms (Islam et al., 2022; Kim H. S. et al., 2021), with rumen microbiota transplantation reducing NCD incidence and frequency by 45.5 and 50.9%, respectively (Bu et al., 2020). Microbial metabolites also show therapeutic potential; comparative metabolomics identified ursodeoxycholic acid as a health-associated marker capable of mitigating colitis and inhibiting ESBL-EAEC (extended-spectrum *β*-lactamase-producing enteroaggregative *E. coli*) infections (He et al., 2022). Furthermore, machine learning enables early NCD prediction (AUC = 84.3%) through biomarker taxa like *Trueperella* (Ma et al., 2020), highlighting the translational value of microbiome profiling.

Despite these advances, the age-specificity of diarrheal dysbiosis remains unexplored. Given the microbiota’s rapid ontogenetic changes, we hypothesize that NCD-associated microbiome perturbations exhibit age-dependent heterogeneity. To test this, we systematically compare fecal microbiomes between diarrheal and healthy calves at three critical stages (1, 21, and 30 days old), with dual objectives: (1) identifying age-stratified diarrheal biomarkers and (2) deciphering dynamic network reorganization patterns. Our findings aim to inform age-tailored microbiome therapeutics, advancing NCD management from empirical treatment to precision modulation.

## 2 Materials and methods

### 2.1 Experimental design and animal management

The study was performed on a commercial dairy farm (>2,500 Holstein cattle) located in Hohhot, Inner Mongolia. Ninety-six female calves born between 1 June and 20 July 2021 were enrolled. Inclusion criteria were birth weight ≥30 kg, serum total protein ≥5.0 g dL^−1^ (DD-2 digital refractometer, MISCO, United States), absence of congenital malformations and no pre-enrolment antibiotic treatment. Calves were housed individually in identical, disinfected hutches (1.2 × 1.5 m) under uniform environmental conditions; all husbandry procedures followed the farm’s standard operating protocols.

### 2.2 Colostrum and milk-feeding program

Colostrum fed to every calf contained 94.06 ± 20.60 g L^−1^ IgG (mean ± SD). Within 1 h of birth, calves received 4 L by bottle; an additional 2 L were given at 8 h post-partum. From day 2 to 30, the whole milk was fed that guaranteed minima of 3.1% fat, 2.8% crude protein, 8.1% non-fat solids, and 11.2% dry matter (DM). Feeding volumes were 3 L twice daily on days 2–7, 4 L twice daily on days 8–21, and 5 L twice daily on days 22–30. Starter feed (DM 86%, crude protein ≥22%, crude fiber ≥10%, ash ≤8.5%, Ca 0.5–2.0%, P 0.4%, lysine 0.8%, NaCl 0.4–1.9%) and clean water were offered ad libitum from day 2 onwards (Supplementary Tables S1, S2).

### 2.3 Health monitoring and diarrhoea diagnosis

Two experienced veterinarians conducted independent daily clinical examinations. Fecal consistency was scored immediately after defecation using the validated 4-point scale of McGuirk (2008): 0 = normal; 1 = semi-formed/pasty; 2 = loose (remains on bedding); 3 = watery (seeps through bedding). Diarrhoea was recorded only when both veterinarians assigned a score ≥2.

### 2.4 Sampling strategy and exclusion

Fecal samples were collected at the first observed diarrhoeic episode occurring at 1, 21 or 30 d of age. Healthy controls were matched to diarrhoeic calves by exact birth date and sampled at the same target age with consistent scores of 0–1. Calves were excluded (*n* = 36) when diarrhoea occurred outside the target ages (*n* = 14), no birth-date-matched diarrhoeic pair was available (*n* = 19), or veterinarians disagreed on the fecal score (*n* = 3).

### 2.5 Sample collection and processing

Mid-stream fecal samples were obtained during spontaneous defecation using sterile spatulas, avoiding ground contact. Samples were snap-frozen in liquid nitrogen within 5 min of collection and stored at −80°C. Total genomic DNA was extracted using the CTAB method (Doyle and Doyle, 1987). DNA integrity was verified by electrophoresis on 1% agarose gels.

### 2.6 Microbial community profiling

The V3-V4 region of the 16S rRNA gene was amplified using universal primers 341F/805R under the following PCR conditions: initial denaturation at 98°C for 1 min; 30 cycles of 98°C for 10 s, 50°C for 30 s, and 72°C for 30 s; final extension at 72°C for 5 min. PCR products were purified using QIAquick PCR Purification Kit (Qiagen, United States) after verification by 2% agarose gel electrophoresis. Libraries were prepared using TruSeq DNA PCR-Free Sample Preparation Kit (Illumina, United States) and sequenced on the Illumina NovaSeq platform (250 bp paired-end reads; Novogene, Beijing).

### 2.7 Bioinformatics and statistical analysis

Raw sequencing data were processed through QIIME 2 (version: 2024.2) pipeline: quality filtering (reads with more than 2 expected errors were filtered out), trimming (Phred score ≥ 25: trunc-len-*f* = 182, trunc-len-r = 220), and denoising using DADA2 plugin to generate amplicon sequence variants (ASVs) (Callahan et al., 2016); taxonomic annotation against SILVA 138 database; and rarefaction to 39,161 sequences per sample for downstream analyses (Bokulich et al., 2018). Alpha diversity was assessed using Shannon index (Wilcoxon test), while beta diversity was analyzed via principal coordinate analysis (PCoA) based on Bray-Curtis distances (PERMANOVA with 999 permutations) (Anderson, 2001). Canonical correspondence analysis (CCA) was employed to examine age and diarrhea effects on microbial composition based on detrended correspondence analysis (DCA) results (axis 1 = 4.20) (Li et al., 2022). Differential abundance analysis was performed using analysis of compositions of microbiomes with bias correction 2 (ANCOM-BC2) (FDR < 0.05) (Lin and Peddada, 2024), with functional potential predicted by PICRUSt2 (Douglas et al., 2020). Microbial co-occurrence networks were constructed using SparCC algorithm (|r| > 0.6, *p* < 0.001) (Friedman and Alm, 2012) and visualized in Gephi 0.9.7 with modularity calculation (Bastian et al., 2009; Blondel et al., 2008). All statistical analyses and visualizations were conducted in R 4.4.2 using phyloseq (McMurdie and Holmes, 2013), vegan (Oksanen et al., 2019), and ggplot2 (Ginestet, 2011) packages.

## 3 Results

### 3.1 Cohort characteristics and fecal consistency profiles

The final cohort comprised 60 calves stratified equally by health status and age, with 30 diarrhoeic and 30 healthy calves distributed across three sampling timepoints (10 per group at 1, 21, and 30 days). Fecal scoring revealed pronounced differences between groups: diarrhoeic calves exclusively exhibited abnormal consistency [57% (17/30) scored 2, 43% (13/30) scored 3], while healthy calves showed normal or mild deviations [87% (26/30) scored 0, 13% (4/30) scored 1]. Age-stratified analysis indicated heightened severity in neonatal diarrhoea, with day-1 diarrhoeic calves displaying the highest proportion of severe cases (60% score 3). Birth weights did not differ significantly between diarrhoeic (36.9 ± 4.1 kg) and healthy (37.8 ± 3.3 kg) cohorts (*p* > 0.05). Complete individual metadata, including fecal scores and morphological descriptions, are cataloged in Supplementary Table S3.

### 3.2 Diarrhea onset age shapes gut microbiota structure in diarrheal calves

**Figure 1:**
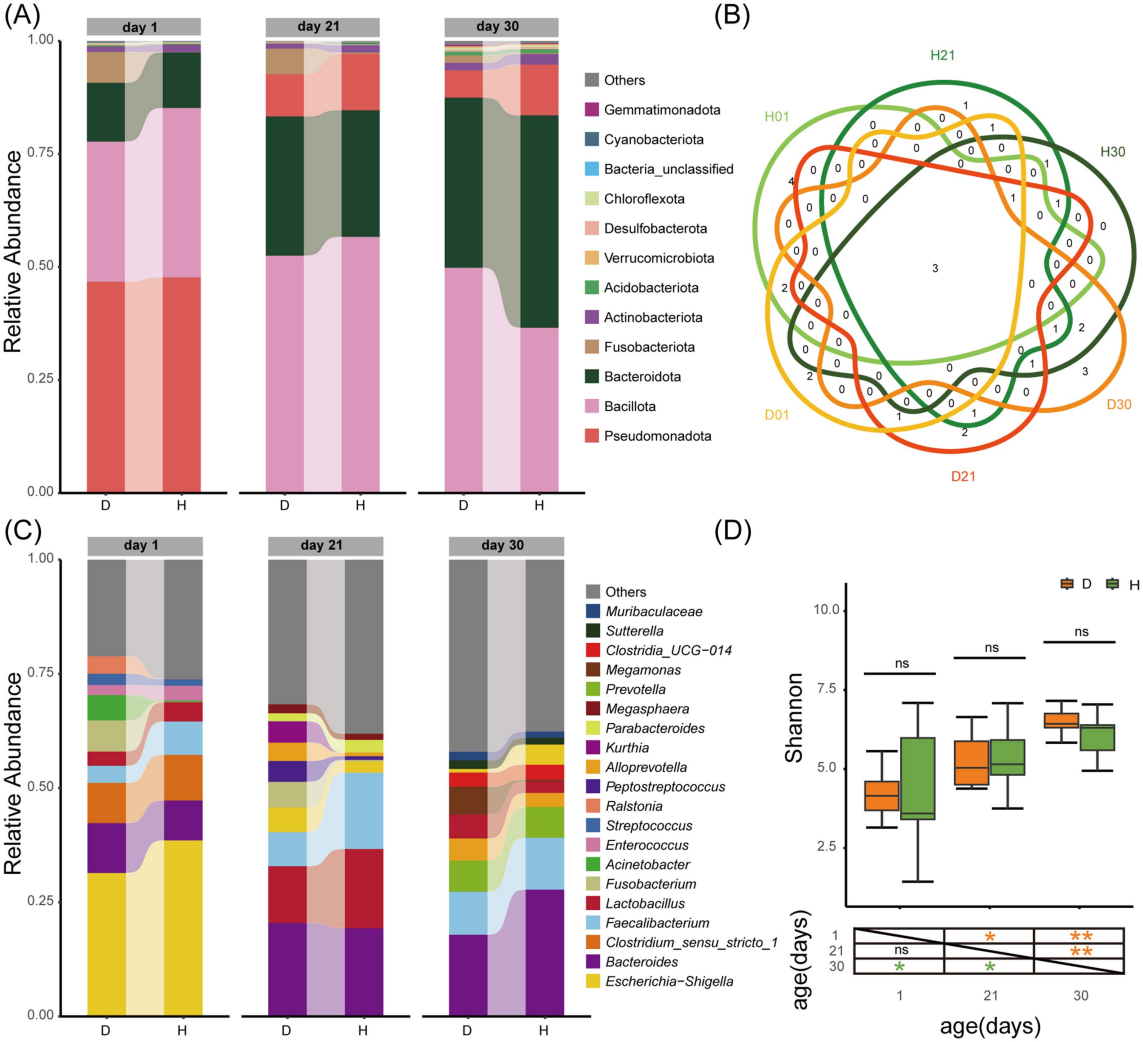
Influence of age and diarrhoea on the gut microbiota diversity and composition of calves. **(A)** Composition of gut microbiota at the phylum level (top 10 in relative abundance). **(B)** Overlap of genus level (top 10 in relative abundance). **(C)** Composition of gut microbiota at the genus level (top 10 in relative abundance). **(D)** Shannon-based analysis of alpha diversity. Group D: diarrheal group (day 1: D01, day 21: D21, day 30: D30); Group H: healthy group (day 1: H01, day 21: H21, day 30: H30). Statistical significance: ns = not significant; *p < 0.05; **p < 0.01.

Sequencing of 60 fecal samples generated 4,075,413 quality-filtered reads (67,923.6 ± 7,129.6 per sample), clustered into 11,112 ASVs representing 64 phyla and 1,613 genera. The phylum-level composition showed dramatic age-dependent shifts (Figure 1A): Pseudomonadota dominated at day 1 (47.23 ± 0.68%), transitioning to Bacillota predominance at day 21 (54.60 ± 2.94%), and finally to co-dominance of Bacillota (43.20 ± 9.40%) and Bacteroidota (42.31 ± 6.63%) at day 30. Genus-level analysis confirmed that age exerted stronger effects than diarrhea status. Genera shared by Diarrheal groups (*Bacteroides*, *Faecalibacterium*, and *Lactobacillus*) constituted the core microbiota present in all groups (Figure 1B). The top 10 genera overlap between healthy and diarrheal calves within the same age group reached 63.33 ± 5.7%, versus only 35.00 ± 7.07% across age group within identical status (Figure 1C). Shannon diversity index increased significantly with age in both healthy (4.20 ± 1.96 to 6.08 ± 0.68, CV: 46.81 to 11.22, *p* < 0.05) and diarrheal groups (4.24 ± 0.77 to 6.58 ± 0.48, CV: 18.12 to 7.33, *p* < 0.01). Crucially, no significant differences between health states at any age (Figure 1D).

**Figure 2:**
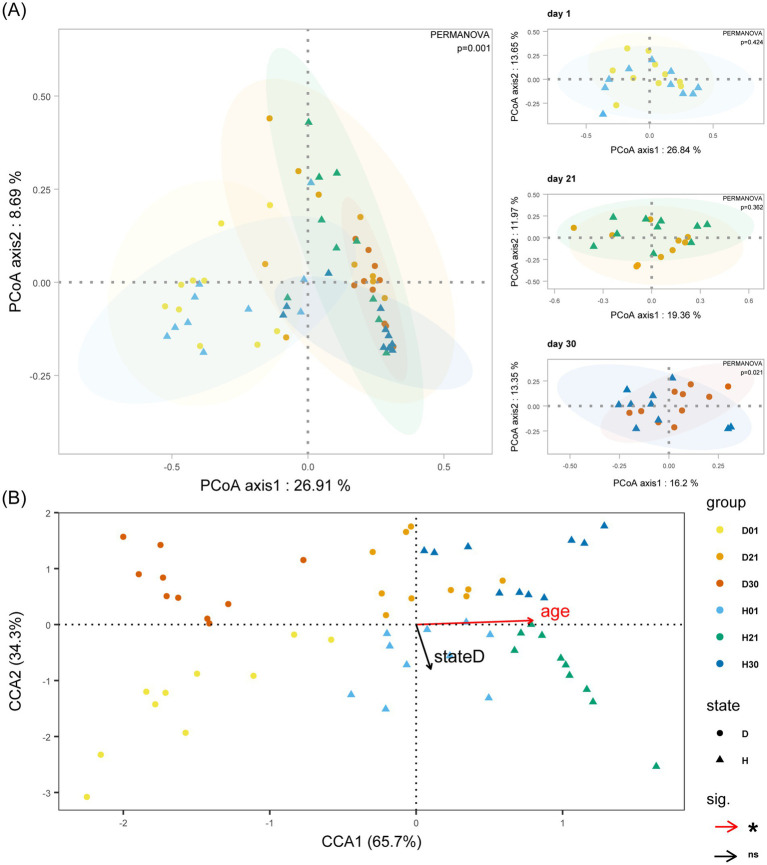
Bray-Curtis distance-based analysis of calf gut microbiota. **(A)** Impact of age and diarrhoea on the gut microbiota beta diversity of calves. **(B)** Impact of age and diarrhoea on the gut microbiota of calves with CCA analysis. Group D: diarrheal group (day 1: D01, day 21: D21, day 30: D30); Group H: healthy group (day 1: H01, day 21: H21, day 30: H30). Statistical significance: ns = not significant; *p < 0.05.

Beta diversity analysis revealed distinct age-stratified clustering patterns in microbial community structure across developmental stages (day 1, day 21, day 30). Significant separation between healthy and diarrheal groups only at day 30 (PCoA, *p* = 0.02; Figure 2A). CCA quantified the independent contributions of age and diarrhea to community variation. Age independently explained 3.68% of variance (*p* < 0.01), while diarrhea explained 1.96% (*p* = 0.14). The near-orthogonal angle (88.1°) between diarrhea occurrence and age vectors suggests non-interactive effects of these factors on calf gut microbiota, indicating statistically independent drivers of microbial community variation (Figure 2B).

### 3.3 Age-specific compositional signatures of diarrhea-associated dysbiosis

**Figure 3:**
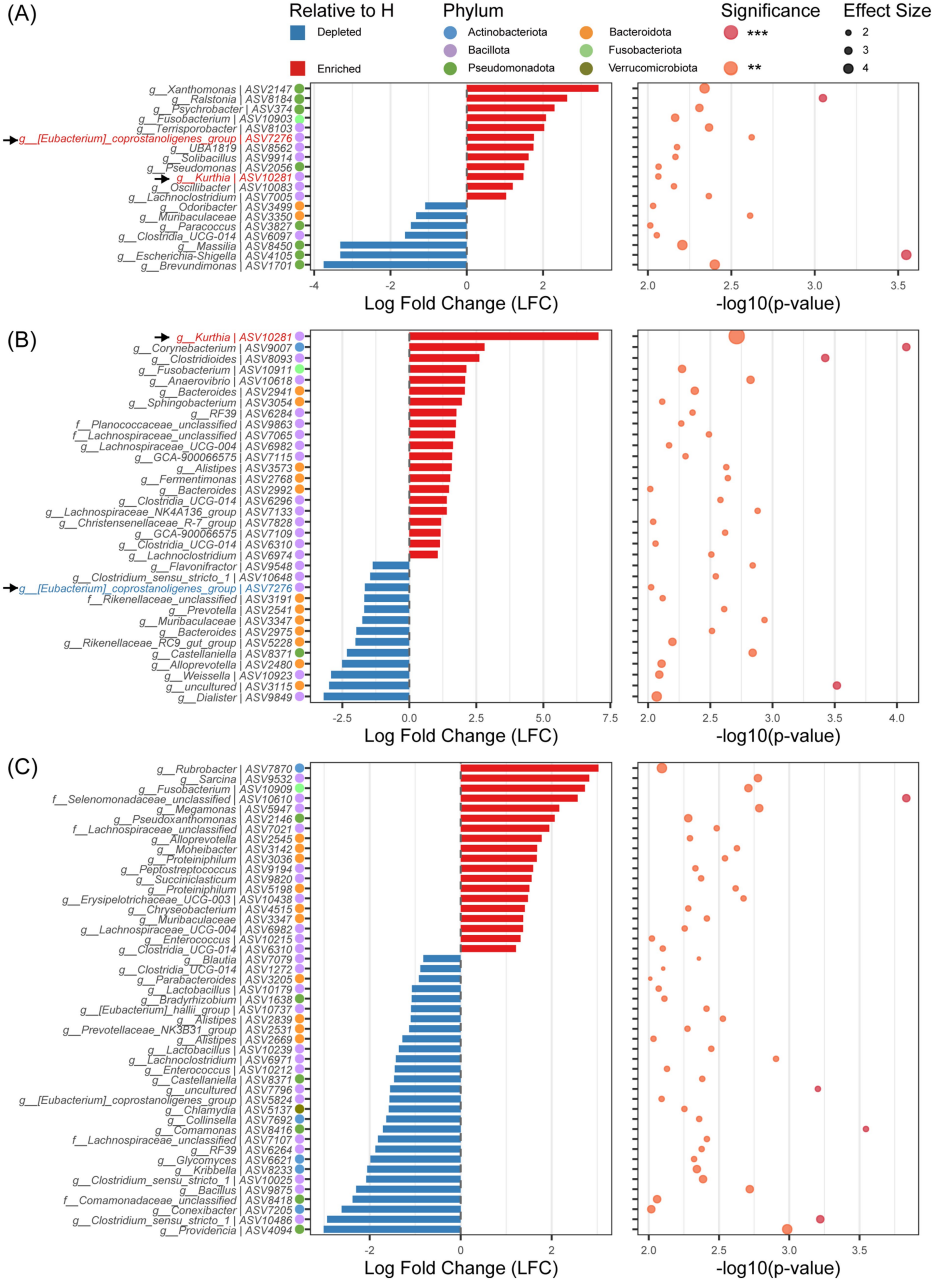
Analysis of differences in ASV levels between diarrhea (D) and healthy (H) at different ages. **(A)** Analysis of differences in ASV levels between D and H at day 1. **(B)** Analysis of differences in ASV levels between D and H at day 21. **(C)** Analysis of differences in ASV levels between D and H at day 30. Statistical significance: **p < 0.01; ***p < 0.001.

Differential abundance analysis of ASV levels (ANCOM-BC2, FDR < 0.001) identified age-specific diarrhea-associated dysbiosis signatures (*p*<0.01, Figure 3): day 1 featured Bacillota/Pseudomonadota shifts (84% of differential ASVs), day 21 showed Bacillota/Bacteroidota alterations (90.5%), while day 30 exhibited complex multi-phylum dysbiosis. At day 1, 19 differential ASVs (19 genera) were identified: 12 enriched genera predominantly from Bacillota (58.33%), Pseudomonadota (33.33%), and Fusobacteriota (8.33%), while 7 depleted genera mainly comprised Pseudomonadota (57.14%), Bacteroidota (28.57%), and Bacillota (14.28%) (Figure 3A). By day 21, 34 differential ASVs (31 genera) emerged: 21 upregulated genera showed Bacillota dominance (66.67%) with emerging Bacteroidota (23.81%), Actinobacteriota (4.76%), and Fusobacteriota (4.76%); 13 downregulated genera were Bacteroidota-predominant (53.84%) (Figure 3B). At day 30, 47 differential ASVs (40 genera) exhibited heightened phylum-level complexity: 19 upregulated ASVs distributed across Bacillota (52.63%), Bacteroidota (31.58%), Actinobacteriota (5.26%), Fusobacteriota (5.26%), and Pseudomonadota (5.26%); 28 downregulated ASVs involved Bacillota (50.00%), Pseudomonadota (17.86%), Actinobacteriota (14.29%), Bacteroidota (14.29%), and Verrucomicrobiota (3.57%) (Figure 3C). Notably, differential bacteria associated with diarrhea showed no consistency across age group. Only two ASVs (ASV7276, *[Eubacterium]_coprostanoligenes_group*; ASV10281, *Kurthia*) showed significant differences in diarrheal group at both day 1and day 21. Specifically, ASV10281 was enriched in the diarrheal group at both day 1 (LFC = 1.49, *p* < 0.01) and day 21 (LFC = 7.06, *p* < 0.01). ASV7276 demonstrated dynamic fluctuations: enriched in diarrheal groups at day 1 (LFC = 1.76, *p* < 0.01), significantly enriched in healthy groups at day 21 (LFC = -1.66, *p* < 0.01), and with no intergroup difference at day 30 (*p* > 0.05; Figures 3A,B).

### 3.4 Age-specific dynamics in gut microbiota function and networks in diarrhea

**Figure 4:**
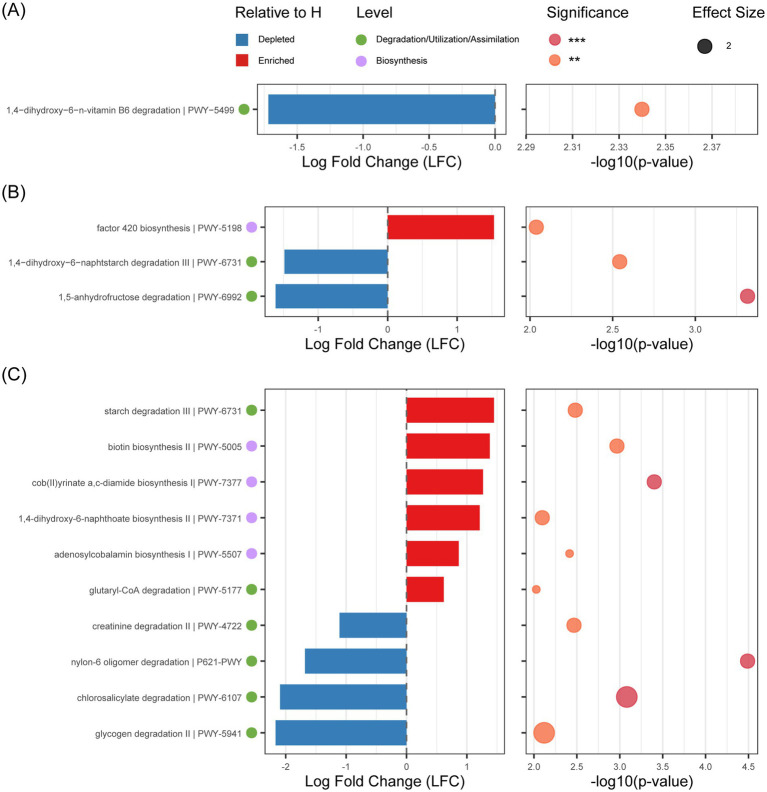
Analysis of differences in the MetaCyc pathway between diarrhea (D) and healthy (H) at different ages. **(A)** Analysis of differences in the MetaCyc pathway between D and H at day 1. **(B)** Analysis of differences in the MetaCyc pathway between D and H at day 21. **(C)** Analysis of differences in the MetaCyc pathway between D and H at day 30. Statistical significance: **p < 0.01; ***p < 0.001.

PICRUSt2 predicted increasing numbers of differentially abundant pathways with age (day 1:1; day 21:3; day 30:10). These primarily affected carbohydrate metabolism and vitamin metabolism (Figure 4). Specifically, starch degradation pathway III (PWY-6731) was enriched in day 21 healthy group (LFC = -1.49, *p* < 0.05) but upregulated in day 30 diarrheal groups (LFC = 1.45, *p* < 0.05).Carbohydrate biosynthesis-related pathways PWY-6992 (LFC = -1.61, *p* < 0.001) and PWY-5941 (LFC = -2.16, *p* < 0.001) showed specific enrichment in healthy calves at day 21 and day 30, respectively. Vitamin metabolism exhibited age-specific alterations. Vitamin B6 degradation pathway (PWY-5499) was significantly reduced in day 1 diarrheal groups (LFC = -1.72, *p* < 0.05), whereas vitamin B12 synthesis pathways (PWY-7377, LFC = 1.27, *p* < 0.001; PWY-5507, LFC = 0.86, *p* < 0.05) were upregulated in day 30 diarrheal groups.

**Figure 5:**
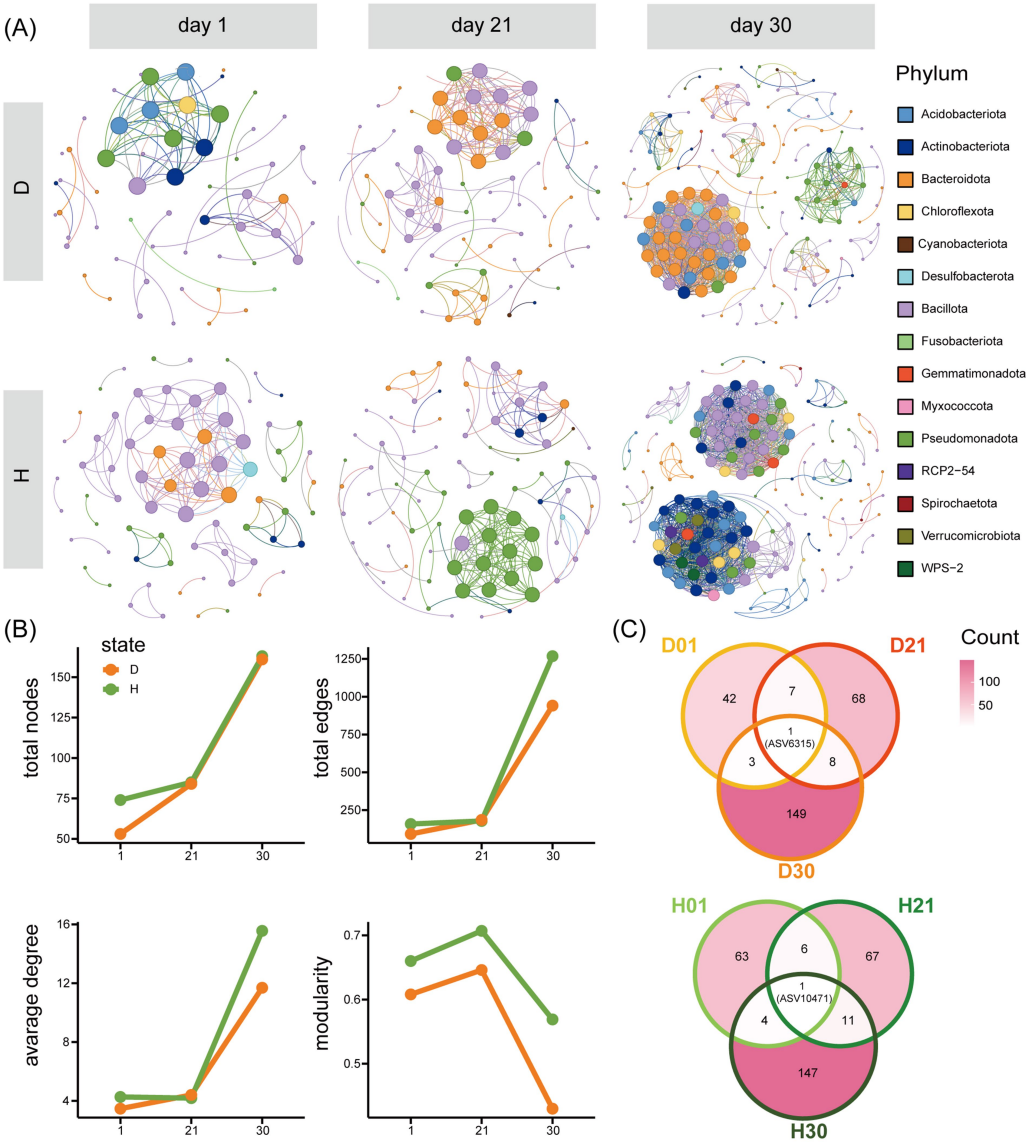
Network analysis of differences between diarrhea (D) and healthy (H) at different ages. **(A)** Network analysis of differences between D and H. **(B)** Network parameter analysis of differences between D and H. **(C)** Overlap of network nodes at different ages in D and H.

Co-occurrence network analysis demonstrated progressive increases in microbial interaction complexity with advancing age, evidenced by substantial expansions in network size (node count increased from 64 to 162) and connectivity (edge number escalated from 125 to 1,105). Diarrheal cohorts exhibited marked reductions in topological parameters, manifesting decreased node counts and diminished network modularity compared to healthy controls (Figure 5B). All networks comprised four core phyla: Actinobacteriota, Bacteroidota, Bacillota, and Pseudomonadota, with Bacillota maintaining dominance across groups. Network composition varied by age and diarrhea status. Diarrheal networks were primarily composed of Bacillota and Bacteroidota, which together accounted for over 60% of the nodes. In contrast, healthy networks evolved from being dominated by Bacillota and Pseudomonadota at days 1 and 21 to a more balanced distribution by day 30 (Figure 5A). Cross-age comparisons revealed unique features (Figure 5C): (1) No shared ASV nodes across all groups; (2) ASV6315 (*Clostridia_UCG-014*) persisted across diarrheal networks but lost interactions in healthy groups; (3) The shared node ASV10471 (*Clostridium_sensu_stricto_1*) in healthy groups exclusively appeared in day 1 diarrheal group. Notably, ASV10281 emerged as a day 21-specific hub (degree = 14), coinciding with its significant enrichment (log2FC = 5.32, *q* = 0.002; Figure 3B).

## 4 Discussion

Our study delineates three key findings about NCD. First, gut microbiota development follows a stronger age-dependent trajectory than diarrhea-induced variation. Second, microbial responses to diarrhea exhibit marked age specificity. Finally, diarrheal dysbiosis progresses from phylum-level imbalances early in life to complex multi-taxa disruptions later. Collectively, these findings substantiate our hypothesis that in neonatal calves experiencing age-dominated gut microbiota succession during the first postnatal month, diarrhea-associated microbial perturbations manifest distinct patterns across different developmental stages. This advances the understanding of host-microbiota interactions and provides a theoretical foundation for microbiota-based intervention strategies against calf diarrhea.

We observed age-dependent succession of gut microbial communities in both healthy and diarrheal calves, which aligns with previously published findings (Kim E. T. et al., 2021; Huang et al., 2024; Du et al., 2023). CCA results revealed that age independently explained 3.68% of microbiota variation, reinforcing the pivotal role of host age in shaping early-life gut microbiota. Interestingly, the microbial impact of diarrhea also exhibited age-specific patterns. 30-day-old diarrheal calves showed distinct microbiota differentiation from the health group (PCoA, *p* = 0.02), while no such divergence occurred in early stages (days 1 and 21). However, whether this divergence is directly driven by an age-specific diarrheal effect remains uncertain. The observed decline in the coefficient of variation for the Shannon index with increasing age suggests an important trend. Prior evidence indicates that inter-individual variation in the calf gut microbiota diminishes over time (Huang et al., 2024; Malmuthuge et al., 2019). Together, these findings imply that high microbial variability during early colonization may obscure diarrhea-associated diversity changes. In summary, although early-life microbial instability may hinder the detection of perturbation signals, the significant community divergence at day 30 supports the hypothesis that diarrhea-associated dysbiosis manifests in an age-dependent manner. Future research should consider larger sample sizes and bioinformatic strategies that account for individual-level variation to better resolve age-specific microbial responses to diarrheal events.

Differential analysis revealed age-specific diarrheal signatures. At day 1, the differentially abundant ASVs in diarrheal calves relative to the healthy group were primarily derived from Pseudomonadota and Bacillota. By day 21, the differences were predominantly driven by Bacillota. While 30-day-old diarrheal calves exhibited co-variation of Bacillota-Bacteroidota. This trajectory aligns with known phylum-level successional patterns during calf gut maturation (Huang et al., 2024; Malmuthuge et al., 2015; Uyeno et al., 2010), suggesting that age-dependent microbial backgrounds may shape diarrhea-related dysbiosis. Importantly, no single ASV was consistently differentially abundant between diarrheal and healthy calves across all three age groups. In contrast, ASV7276 (*[Eubacterium]_coprostanoligenes_group*) demonstrated a distinct age-dependent abundance pattern. It was significantly enriched in the diarrheal group at day 1 but shifted to enrichment in healthy calves at day 21, with no significant difference observed at day 30. The group is known to generate short-chain fatty acids (SCFAs) and potentially interact with sphingosine to maintain the host lipid homeostasis (Si et al., 2018; Wei et al., 2021). Additionally, serum metabolomic analysis of diarrheal calves revealed significant elevations in SCFAs such as 2-methyl-3-hydroxybutyric acid and coordinated alterations in fatty acid biosynthesis pathways (Huang et al., 2020). However, the mechanisms underlying the dynamic shifts of *[Eubacterium]_coprostanoligenes_group* across age-stratified diarrheal calves remain to be elucidated.

At the functional level, diarrheal calves showed significant alterations predominantly in carbohydrate metabolism and B-vitamin biosynthesis pathways, which also exhibited age-specific response patterns. No function pathway was universally differentially abundant across all three age groups when comparing diarrheal and healthy calves. It is worth noting that PWY-6731 (starch degradation pathway III) showed the age-stratified abundance dynamics: no intergroup difference was observed on day 1, the healthy group showed significant enrichment by day 21, and this trend reversed with the diarrheal group becoming enriched by day 30. The reversal in enrichment pattern may reflect complex interactions among gastrointestinal development, evolving dietary inputs, and diarrhea onset. Further investigation is required to elucidate the underlying regulatory mechanisms.

Co-occurrence network analysis revealed increasing microbial interaction complexity with age, diarrhea reduced network complexity, consistent with intestinal homeostasis disruption (Kwon et al., 2021; Ku et al., 2025). The persistent presence of ASV6315 (*Clostridia_UCG-014*) in diarrheal networks, coupled with its reported pro-inflammatory associations (Brandsma et al., 2019; Wang et al., 2021), suggests its potential role in diarrhea-associated microbiota remodeling. The day 21-specific emergence of ASV10281 (*Kurthia*) as both a differentially abundant taxon and network hub suggests this oxygen-tolerant bacterium (Mukhopadhyay et al., 2019; Guo et al., 2016) may play an ecological role in mid-lactation diarrhea. These findings provide novel insights into the dynamics of microbial stability during diarrheal pathogenesis.

Methodologically, our multi-angle approach—combining differential abundance, functional prediction, and network analyses—provides a systems-level view of diarrheal dysbiosis. However, limitations include sample size constraints and the inherent resolution limits of 16S sequencing. Future studies should employ longitudinal designs with macrogenomics, metabolomics and *in vitro* culture experiments to systematically elucidate the pathological regulatory mechanisms of key species (e.g., *Kurthia*, *Clostridia_UCG-014*), the causal relationship between temporal sequencing of colony development and diarrhoea susceptibility, as well as the potential for application of age-specific microbial markers.

## 5 Conclusion

This work establishes that gut microbial responses to diarrhea in neonatal calves are fundamentally age-dependent, progressing from simple phylum-level shifts to complex multi-taxa disruptions as the microbiota matures. These findings advance our mechanistic understanding of NCD pathogenesis and highlight the need for age-tailored microbiome interventions.

## Data Availability

The raw sequencing data generated in this study are publicly available in NCBI Sequence Read Archive (http://www.ncbi.nim.nih.gov/sra) under the accession number PRJNA1263132.
